# Impact of COVID-19 on vegetable supply chain and food security: Empirical evidence from Bangladesh

**DOI:** 10.1371/journal.pone.0248120

**Published:** 2021-03-05

**Authors:** G. M. Monirul Alam, Most Nilufa Khatun

**Affiliations:** 1 Bangabandhu Sheikh Mujibur Rahman Agricultural University, Gazipur, Bangladesh; 2 University of Southern Queensland, Toowoomba, Australia; Neijiang Normal University, CHINA

## Abstract

In Bangladesh, the COVID-19 pandemic is likely to have substantial effects on the livelihood of people, but smallholder vegetables growers will be even more affected because of the perishability nature of the product. The first case of COVID-19 was confirmed in Bangladesh on 8th March, 2020 and consequently the country went into lockdown on 26 March, 2020. This study has made a survey of vegetables farmers through a mobile phone to understand the impact of COVID-19 on vegetables supply chain, gross margin and the future production plan of the growers. In Bangladesh, the lockdown has disrupted the food supply chain and increases the likelihood of food insecurity. Lockdown has impeded vegetable farmers’ access to markets, thus limiting their productive and sales capacities. The price of yield has dropped by more than half resulting in huge loss for vegetable growers. The loss incurred by the farmers for producing Brinjal, Cucumber, Pointed gourd, Yardlong beans and Bottle gourd are BDT 4900, BDT 10900, BDT 57400, BDT 52500 and BDT 18500 per acre respectively as a result of COVID-19. The decreased income increases farmers’ likelihood of vulnerability and food insecurity and poses a challenge to continued produce. ‘Cash support’ is more important than ‘food support’ in order to keep vegetable farmers in farming, to ensure a ready supply of necessary low-cost resources, and to help fight against the upcoming food shortage.

## Introduction

The coronavirus disease (COVID-19) caused by a virus named ‘SARS-CoV-2’ has created an unprecedented situation globally. It was first identified in Wuhan City, Hubei Province of China in the late December 2019 [1]. The ‘COVID-19’ pandemic has posed a grave menace to human health, economy and food security both in developed and developing countries [2–4]. However, the poor people in developing countries such as Bangladesh will be impacted disproportionately mainly due to their poor income and inadequate healthcare system [5]. The ‘COVID-19’ pandemic might have longer effects on income of poor people like smallholder vegetables farmers’ [6] and consequently on their food security and nutrition [7]. The ongoing rapid human to human transmission of COVID-19 is a big threat for a populous country Bangladesh (1265 persons per km^2^) where a large number of people have to live in a single room and/or in street and/or slum.

Bangladesh is predominantly an agrarian economy where most of the poor people live in rural areas and reliant on agriculture for their livelihood and food security [8]. The COVID-19 pandemic also attracted Bangladesh which was first confirmed on March 8, 2020, consequently the country went into lockdown on March 26, 2020 [9]. This arises at a time when smallholder farmers were gearing up the harvest of rabi crops. The sudden lockdown has led to the disruption of supply chain including vegetables supply chain which is high value perishable products.

In fact, Bangladesh is suitable for producing various vegetables (more than 142 types of home-grown and exotic vegetables produced in the country) due to fertile land and environment [10]. Bangladesh retained 3^rd^ position in global vegetables production [11]. Vegetables is grown in both winter (mid-December to mid-February) and summer (mid-April to mid-June) seasons in the country. The country has experienced tremendous growth of vegetables production over the last couple of decades mainly due to the higher economic return from vegetables production [12–16] along with the development of improved seeds and technologies [10]. Over the last five years (2013/14 to 2017/18) the production of vegetables has increased by about 35% in the country. Whereas, the area under vegetables production has increased from 9.68 lac hectare (ha) in 2013/14 to 11.69 lac ha in 2017/18. In Bangladesh, about 16.2 million farm households are involved with vegetables cultivation covering an area of about 2.63% of the total cultivable land [17].

A large number of farmers mainly smallholder farmers (farm households that own or/and cultivate just 0.05–2.49 acres of land) are producing vegetables commercially in Bangladesh [10,18]. They are able to expand its chain from local to export markets. Rapid urbanization and increase of income have also contributed to increase the demand of vegetables consumption in Bangladesh. But due to COVI-19 pandemic, the supply chain of vegetables has broken down resulting huge loss to the growers. Though stimulus packages for agriculture have been announced, they fail to offer clear incentives for smallholder vegetable farmers who are incurring huge loss from their current produce. This has increased the likelihood of vulnerability and food insecurity of vegetables growers. Based on a survey of vegetable growers and other key informants, this study aims to understand the impact of COVID-19 on vegetable supply chains, gross margins, food security, and growers’ future production plans.

The paper is structured as follows: the section 2 describes the methodology of the study; results and discussion are presented in section 3 including the COVID-19 impact on food security and future production plan; and section 4 offers the conclusion and some policy implications of the study.

## Methodology

### Study area and sample size

**Fig 1:**
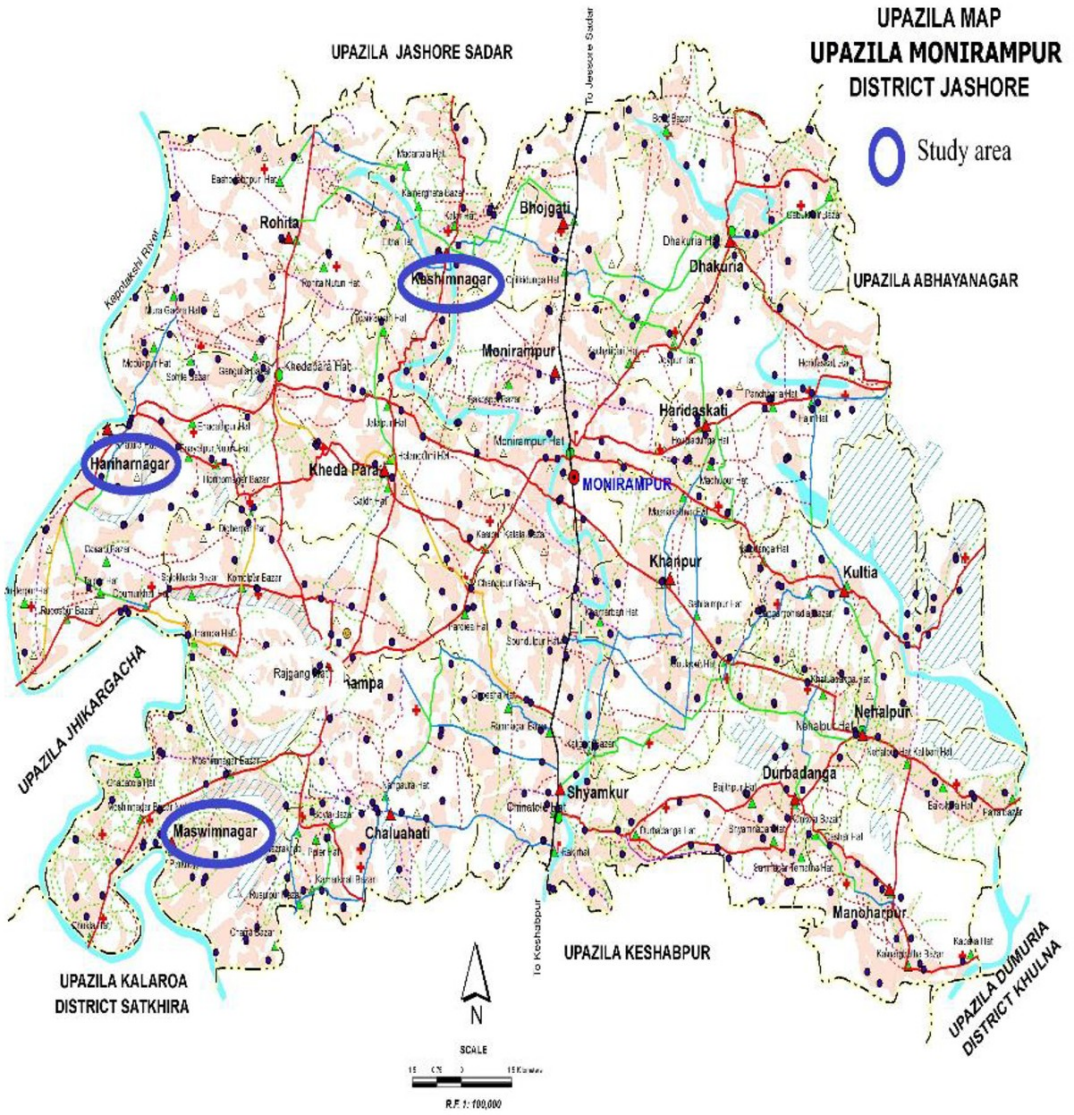
The study area: The Monirumpur Upazila, Bangladesh.

Actually, a detailed survey was conducted in the Monirampur upazila (Fig 1) of Jashore district between January and February 2020 to assess the market participation of the smallholder vegetables growers. The study area is a very important vegetables growing area in the country. They used to export vegetables in the overseas market. The list of vegetables growers was collected from the Department of Agricultural Extension [17]. Using statistical formula, we conducted a face-to-face interview of 120 respondents from five villages (Horishpur, Hakimpur, Rampur, Sahapur, and Rosulpur) through a structured survey questionnaire. Household head was the survey respondent. The study covered more than 15% of the study population.

### Data collection

After the COVID-19 pandemic, a mobile phone interview of 100 vegetable farmers from 120 farmers was conducted from 8 June to 14 July, 2020. Most of the farmers (98%) had ownership of a mobile phone. Both qualitative and quantitative data were collected for this study. The mobile interview contains information on the marketing channel, current price of the product, food security and future production plan. Information was collected for five vegetables; brinjal, cucumber, pointed gourd, yardlong beans and bottle gourd. Interviews were conducted by phone and lasted about 15 to 20 minutes. Moreover, the key informants’ interview was also conducted through a mobile phone. The underlying purposes of the discussions were to obtain views on COVID-19 pandemic and vegetables supply chain.

### Analysis of data

Gross margin (GM) analysis was performed to estimate the profitability of vegetables production based on two scenarios: (i) business as usual case (normal situation), and (ii) COVID-19 pandemic case. For short run analysis as well as for the farm planning GM analysis is widely used [19]. Gross margin analysis gives an estimate of the difference between total return and variable cost (TR- VC). Fixed costs are ignored as these are common to all activity options.

#### Gross return

Gross return was calculated by multiplying the total volume of production of an enterprise by the average prices of that product in the harvesting period. The following equation was used to calculate gross return:

ΣGR=ΣQm.Pm

Where, GR = Gross return, Qm = Quantity of product, Pm = Per unit price

GM=ΣGR–ΣPxiXi

Where, Xi = Quantity of the ith variable input, Pxi = Per unit price of the ith variable input.

### Ethical standard

The ethical standard was maintained during the research. The study was approved by the research Management committee (RMC) of Bangabandhu Sheikh Mujibur Rahman Agricultural University. Before each interview, the research purpose and the confidentiality of the data were described, and then their verbal consent to provide information voluntarily were taken. The questionnaire content and procedure were properly reviewed by the research team.

## Results and discussion

### Livelihood conditions

The information on household socio-demographic and economic characteristics can be served as the delimitation of the study so that whatever findings or outcomes derived from this study can be described within the domain of this profile [20]. The major socio-economic characteristics of the respondents are discussed below.

Average age of the respondent was around 44 years. Most of the smallholder vegetables growers were middle age group belong to 31 to 60 years of age. Currently, the life expectancy at birth in Bangladesh is 70.3 years [21]. The average family size was 5.21 which is comparatively larger than national average of 4.5 [21]. The average education level of the respondents was below primary level: 3.17 years of schooling. However, 30% of respondents did not attend school. Only 13% had more than secondary education level (Table 1).

**Table 1:** Socio-economic characteristic of the smallholder vegetables growers.

Characteristics/Variables	Number	Percentage
*Age of HH head*	*(Mean*: *44; Range*:*25–65)*	
≤30 years	5	4
31–45 years	51	43
46–60 years	57	47
61–65 years	7	6
*HHs family members*	*(Mean*: *5*.*21; Range*:*3–8)*	
3	23	19
3–5	79	66
≤ 6 members	18	15
*Education*	*(Mean*: *3*.*77 years; Range*: *0–16)*	
Illiterate	24	20
Primary (level 1–5)	49	41
Secondary (level 6–10)	23	19
Higher secondary (level 11–12)	14	12
˂ Higher secondary (level 12–16)	10	8
*Employment status*		
Agriculture	97	80
Business + Agriculture	14	12
Services + Agriculture	9	8
*HHs yearly income (BDT)*		
≥ 50,000	4	3
50, 000–100,000	25	21
10,001–150,000	68	57
≤151,000	23	19
*Farm category*	*(Average farm size*: 0.64 acres*)*	
Large farm (>7.49 acres)	3	3
Medium farm household (2.5–7.49 acres)	6	5
Small farm household (0.5–2.49 acres)	87	73
Landless (<0.5 acres)	24	19

Note: Household = HH.

The average farm size was 0.64 acres where only 2% and 6% were large (>7.49 acres) and medium farmers (2.5–7.49 acres) respectively. Most of the farmers (97%) relied on agriculture for their livelihood. The average annual income of the farmers estimated at BDT 76500 of which about 53% came from vegetables income (Table 1).

Vegetable farmers were found to maintain a hygiene sanitation system, drinks tube well water, have access to modern amnesties like electricity. Few of them (10%) are found to practice homestead/backyard gardening. Around 35% respondents were found to visiting to the MBBS doctors in upazila level or district level, 45% rely on village doctors and 20% rely on both depending on the situation. A small percentage of people (24%) are member of either cooperative society or farmer’s group. Farmers mainly use vans, bicycles, rickshaws, scooters, and tempo driven by small machines to market their vegetables [22].

### Comparison of vegetables supply chain

Supply chain can play a vital role in stimulating production and also in accelerating the pace of economic development in a country. Vegetables is a high value perishable crop and farmers usually do not store it for getting the higher price in future. Vegetables is usually delivered through a number of channels but due to lockdown as a result of COVID-19 pandemic, the supply chain has been disrupted. Therefore, vegetables supply chain is discussed based on two scenarios: (i) business as usual case (normal situation), and (ii) COVID-19 pandemic situation.

#### Business as usual case

In the study area farmers usually sell their products in the nearest markets or cell centre. Due to the intensity of vegetables production in the area, a large number of middlemen and traders from outside the areas were found to functioning there resulted in a growing demand for the produce [23]. They have extended their linkages to both the urban and overseas markets.

**Fig 2:**
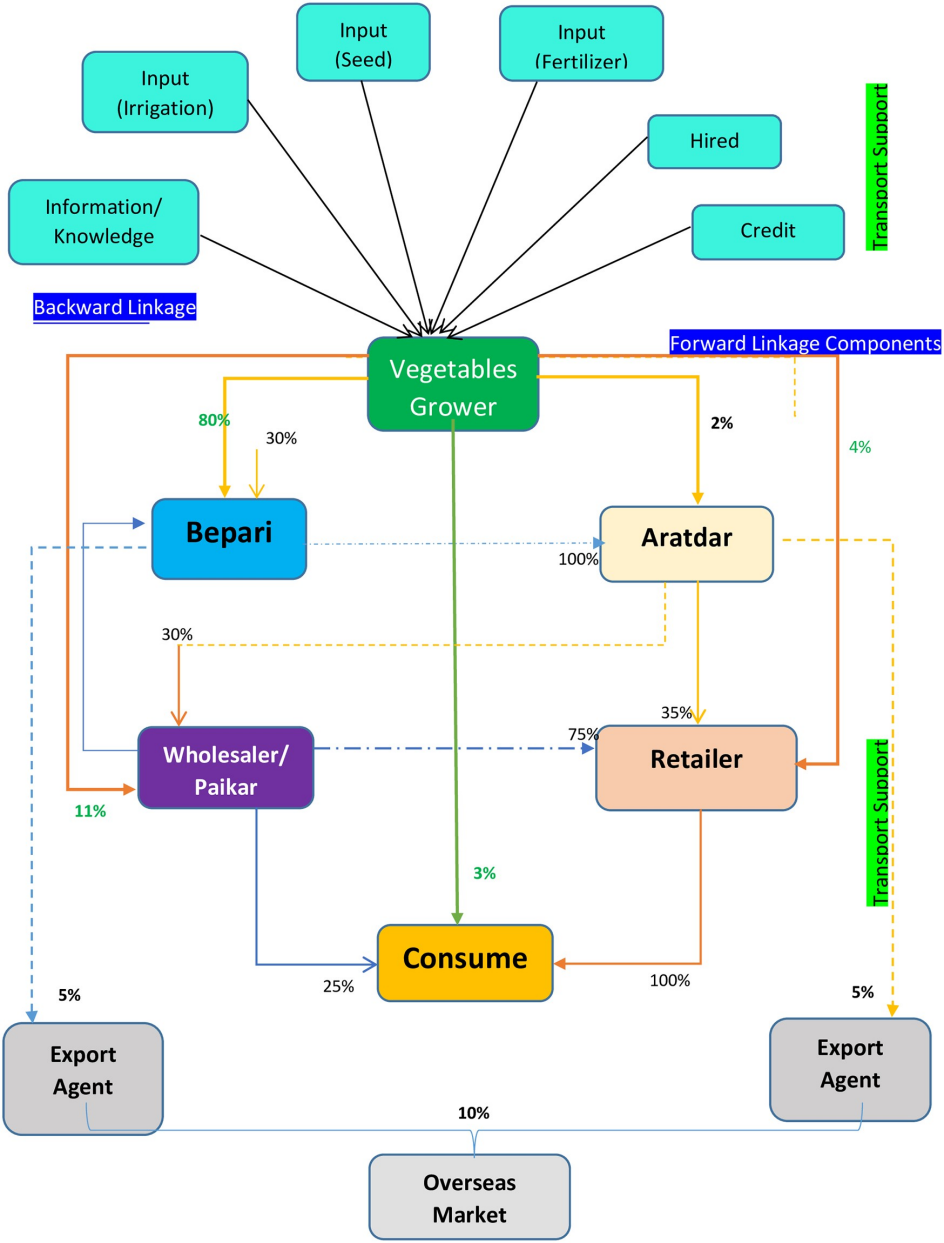
Supply chain of vegetables in the study area (Business as usual case).

The supply chain varies from vegetables to vegetables to some extent. However, a typical vegetables supply chain in the study area is presented in Fig 2. The backward linkage actors (input suppliers) play a crucial role in stimulating vegetables production in the country. The main intermediaries in the vegetables market in the study area were *bepari* (buy from farmers and sell paikar and retailer through aratdar), *aratdar* (act as a commission agent in big or wholesale market with permanent staffs and establishment), wholesaler/*paikar* (usually they buy in bulk volume), and retailer (sell directly to the consumers). Bepari is the most important market actor of vegetables supply chain in the study area. They purchase from the farmers, sale mostly to aratdars and a small amount to the export agents, if they are contacted for exporting in the European market (not more than 5%). In the study area, aratdar sells to wholesaler, beparies of remote or different upazilas, and often some retailers come to purchase vegetables from aratdar (Fig 2).

#### Flow of price information

Farmers usually get price information from the traders and local markets. They do not usually verify the market price while most of them (about 98%) have mobile phone ownership. They did not receive any market information through mobile phone but expressed interest to get price/market information of their products through a mobile phone.

#### COVID-19 pandemic situation

**Fig 3:**
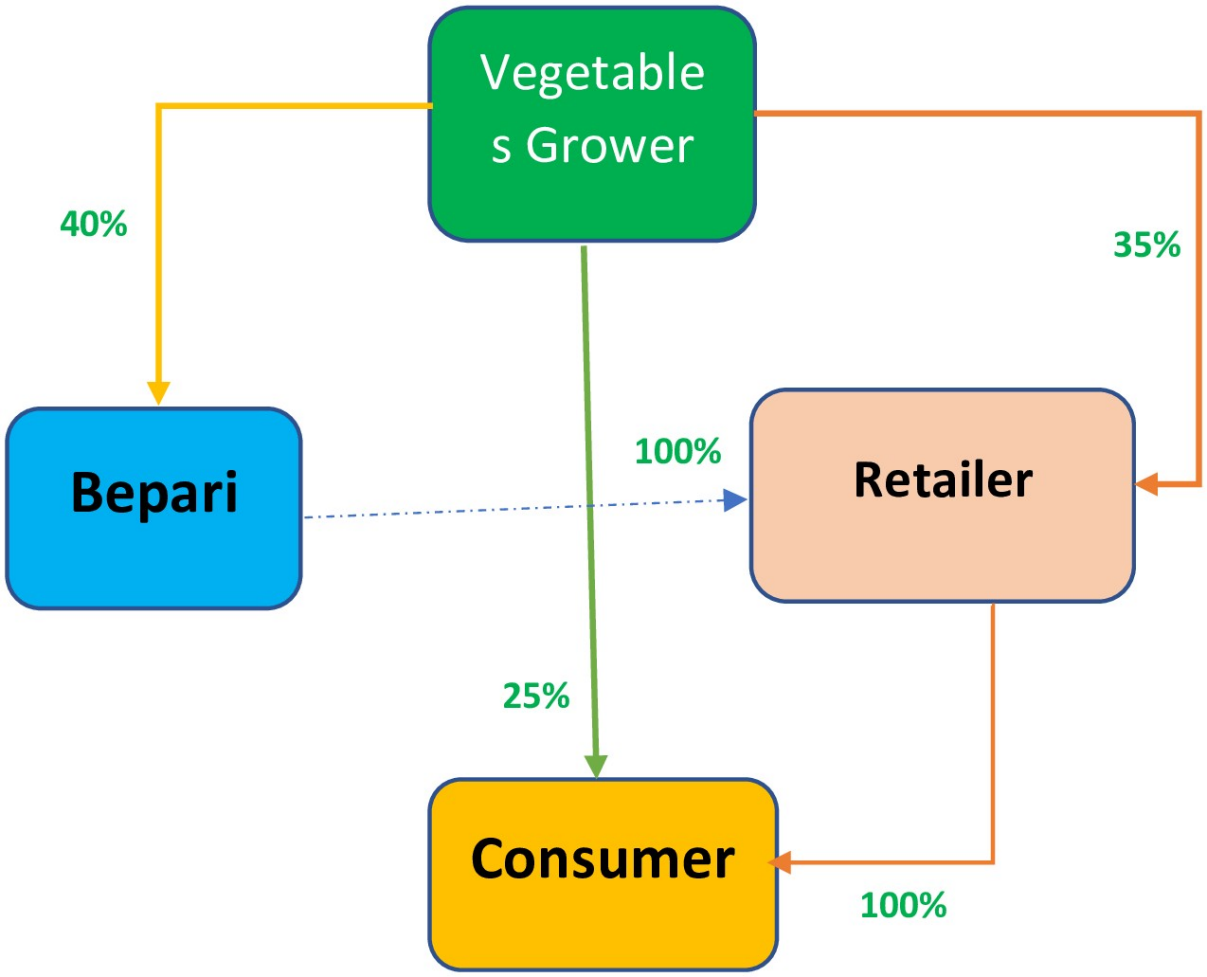
Supply chain of vegetables in the study area during COVID-19.

The COVID-19 pandemic has caused the breakdown of vegetables supply chain (Fig 3). Due to movement restrictions, traders (bepari and others) were not able to come in the area. Farmers were forced to sell some parts of their product (about 25%) in the local markets directly to the consumers. Local baparies were available to the local markets, albeit very limited in number, but unable to send vegetables to distance urban and overseas markets due to lockdown. Respondent opined that reduced purchasing power of the urban consumers due to job loss has contributed greatly to fall the demand of vegetables [24]. On the other hand, vegetables growers have remarkably increased vegetables consumption and distribution among relatives and friends. All these contributed to decline the price of vegetables dramatically resulted in loss of vegetables growers discussed in the next section.

### Cost and return of vegetables production

Smallholder vegetables growers usually employ family labour to perform huge production and harvesting related activities. This is one of the major benefits of producing vegetables for the smallholder farmers. However, this analysis employed the principles of opportunity cost of farmer having to hire the labour. The usual labour cost per day was BDT 400 (Bangladesh Currency: US$1 ≈ 83 BDT as of 25 April, 2020) in the area.

Though production of vegetables varies across farmers, land types, soil fertility but price of yield is the main contributing factor of profitability. In normal times, the average output price per quintal for Brinjal, Cucumber, Pointed gourd, Yardlong beans and Bottle gourd would be BDT 22000, BDT 25000, BDT 20000, BDT 28000 and BDT 20/pic respectively (Table 2).

**Table 2:** Comparison of cost and return between business as usual case and COVID-19.

Cost Items	Pointed gourd (BDT)	Cucumber (BDT)	Bottle Gourd (BDT)	String beans (BDT)	Brinjal (BDT)
Land preparation and seed	11400	9500	6900	7500	9000
Fertilizer	22500	9000	15000	12000	22200
Insecticides	28500	9000	9000	19500	55200
Irrigation, macha and others	48000	33000	51000	49500	43500
Intercultural operation and harvesting	57000	16000	15000	46500	15000
Variable cost	167400	76500	96900	135000	144900
*Gross Return*					
Yield (ton/acre)	10	6.47	11200	7.5	14
Price/ton (Business as usual)	20000	25000	20/pic	28000	22000
Price/ton (During COVID-19)	11000	10000	7/pic	11000	10000

Source: Field Survey, 2020.

The estimated average variable cost for producing Brinjal, Cucumber, Pointed gourd, Yardlong beans and Bottle gourd were BDT 144900, BDT 76500, BDT 167400, BDT 135000 and BDT 96900 per acre respectively. Due to COVID-19 pandemic, price of yield per ton were found to reduce more than double. Therefore, the estimated gross return per acre were BDT 140000, BDT 64700, BDT 110000, BDT 82500 and BDT 78400 for Brinjal, Cucumber, Pointed gourd, Yardlong beans and Bottle gourd respectively. Whereas, in a normal time, the gross return would be BDT 308000, BDT 161750, BDT 200000, BDT 210000 and BDT 224000 for Brinjal, Cucumber, Pointed gourd, Yardlong beans and Bottle gourd respectively, *ceteris paribus*. Cucumber incurred the lowest variable cost. Return per Taka investment (variable cost basis) were 2.12, 2.14, 1.2, 1.6 and 2.3 respectively for these vegetables, compared to 0.96, 0.85, 0.65, 0.61 and 0.80 respectively during a normal year.

### Comparison of gross margin

**Fig 4:**
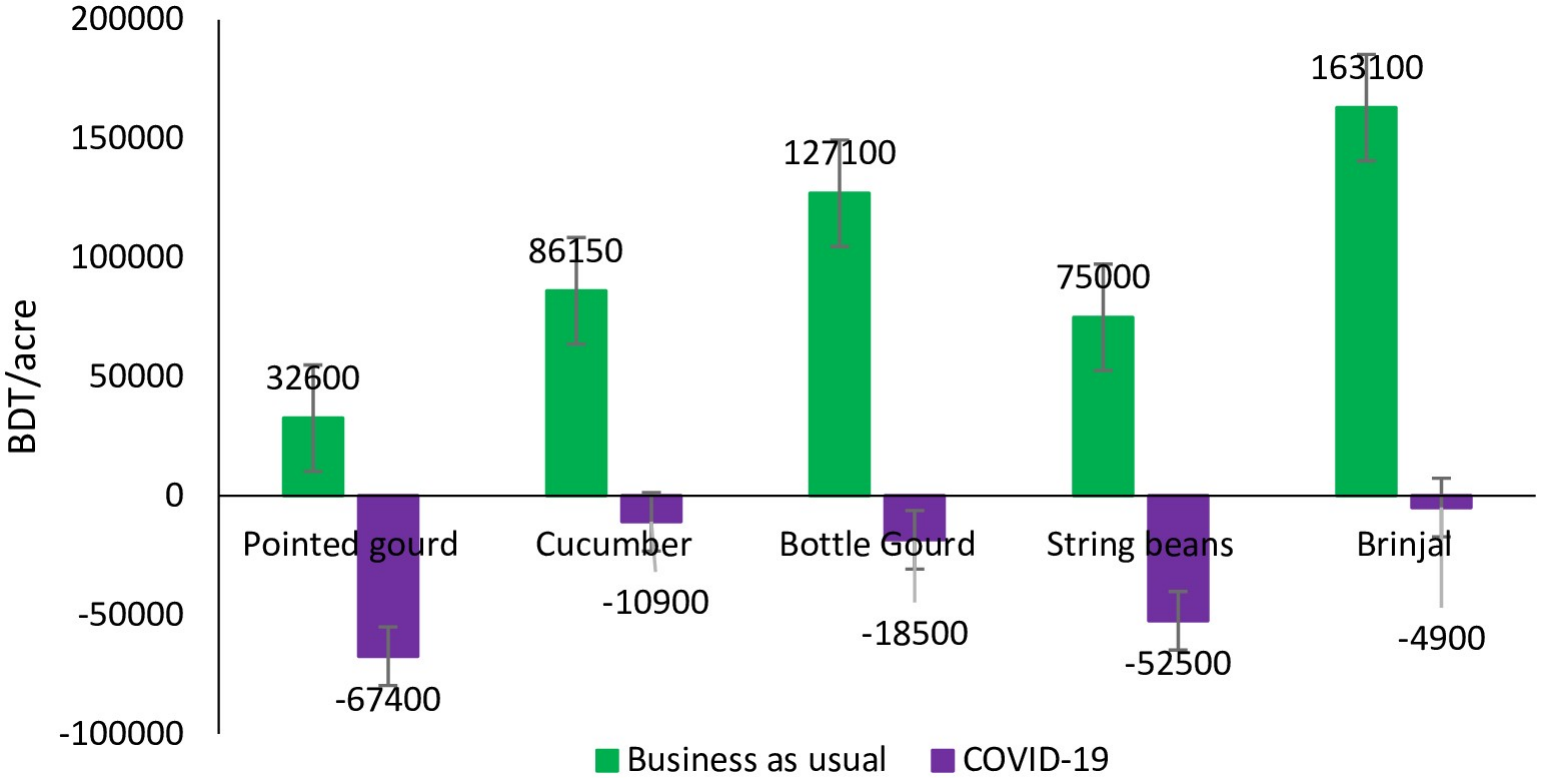
Comparison of gross margin between COVID-19 and business as usual case.

Gross margin (GM) analysis indicates that smallholder vegetables growers incurred substantial loss due to COVID-19 pandemic (Fig 4). Farmers opined that consumption of vegetables has declined both in urban and rural areas due to drastic income fall. The confluence of lockdown and income fall caused a huge loss of the vegetable’s growers. Based on price information provided by the farmers, the estimated loss for producing Brinjal, Cucumber, Pointed gourd, Yardlong beans and Bottle gourd were BDT 4900, BDT 10900, BDT 57400, BDT 52500 and BDT 18500 per acre respectively due to COVID-19, *ceteris paribus*. In a normal time, they would get the return of BDT 163100, BDT 86150, BDT 32600, BDT 75000 and BDT 127100 per acre for producing Brinjal, Cucumber, Pointed gourd, Yardlong beans and Bottle gourd respectively. There were, however, a difference in the gross margin between the respondents (Standard Deviation was BDT 12360/acre) mainly due to yield difference which indicates the necessity of training to improve farmers’ efficiency to reduce the yield gap.

### Impact on food security and future production plan

The COVID-19 has jeopardised the life of the farmers. The surveyed farmers are greatly reliant on income from vegetables (about 53% of their income comes from vegetables) for their survival and continue farming. Though they are impacted by COVID-19 in different ways but dwindling income is the most significant one which ultimately led them to reduce consumption. Most of the telephone surveyed farmers are found concerned about their future food security. Many of them have already reduced the number of meals per day (usually they took meals three times a day) and the majority of them are forced to reduce food items such as purchasing fish and meat. As one respondent stated:

*‘Our livelihood is highly dependent on income from vegetables*. *The adult members of the family are bound to reduce the number of meals (usually three meals a day) due to a drastic decline of income from vegetables’*

*(Interviewee #5)*.

Another commented:

*‘We are now purchasing the necessary food items only*. *Due to the shortage of income we are leaving purchasing protein-rich foods such as fish and meat*. *Our main focus on to start the new production processes’*

*(Interviewee #17)*.

Though farmers are impacted by COVID-19 in many ways, dropping income appears to have had the most damaging effect. Poor households are adjusting to income shocks by consuming less nutritious food and more cereals, and turning to public food relief programs for their survival [25]. A prolonged consumption of less nutritious food might make them prone to ill health/sickness which may ultimately lead to missed work and a further cycle of poverty. Farmers also expect to face challenges in purchasing inputs due to income loss. Cash is needed immediately to enable them to smoothly start further production. One farmer explained why cash was currently more important than food support:

‘*Without cash money we cannot purchase seed, fertilizer and other necessary inputs to start the production process for the next season*’

*(Interviewee #47)*.

Though most farmers were concerned about food security, prices and distribution, they also tended to be optimistic about their capacity to increase food production. COVID-19 might slow down the increasing urbanization rate in Bangladesh due to income fall and raising food price [26]. Many suggested they would return to farming to support themselves which in turn would contribute to increase food production and availability. As one farmer put it:

‘*My son comeback from the capital of Dhaka due to a job lose as a result of COVID-19 pandemic. His participation in farming might help me to produce more vegetables profitably since vegetables production is labour-intensive work*’

*(Interviewee #65)*.

We acknowledge the *caveat* of this study that vegetables production loss is estimated based on output price only. There might be other reasons for vegetables growers’ loss such as changes in yield, input price, etc. But price is the important determinants of profit/loss of any enterprise. As one respondent stated:

‘*Price fall due to COVID-19 was the main reason of incurring loss from vegetables productions which affecting our food security’*

*(Interviewee #28)*.

## Conclusions and policy recommendations

The COVID-19 pandemic is predicted to create a food crisis globally. Bangladesh is—an agrarian economy—affected by ‘COVID-19’ pandemic which was officially declared on 8 March 2020, and consequently the country went into lockdown on 26 March, 2020. Though all walks of people are affected due to COVID-19, however, perishable crops such as vegetables’ producers are affected severely because of the breakdown of supply chain. Vegetables form a major share of their family income. Lockdown impede the farmers’ access to market limiting their productive capacities and selling their produce. Due to the collapse of supply chain of vegetables, the estimated loss for producing Brinjal, Cucumber, Pointed gourd, Yardlong beans and Bottle gourd are BDT 4900, BDT 10900, BDT 57400, BDT 52500 and BDT 18500 per acre respectively. The lower income from vegetables forced farmers to reduce the number of meals and food items per day and poses a huge challenge to continue produce.

Government humanitarian assistance programmes such as food support do not adequately ensure farmers’ survival into the next production cycle. Therefore, additional financial support (cash support) is important to keep smallholder vegetable growers in farming and to maintain the fight against the COVID-19 pandemic. Seed and other input supplies should be ensured. The movement of seasonal migrant farmers should be supported through freer access to medical facilities. The coverage of ongoing social safety net program must be strengthened in order to safeguard the food security and livelihood of vulnerable people. Information on reserves and market situations should be provided. Appropriate steps should be taken for supporting homestead/backyard gardening as an important source of family nutrition and income supplement.

Quick end to the COVID-19 pandemic is unlikely suggesting a long-term impact on people’s livelihood and economy of Bangladesh like global economy. In the absence of a COVID-19 vaccine, awareness programmes promoting social distancing and adherence to WHO guidelines regarding hand washing and wearing a mask should be emphasized as keys to controlling the spread of the disease. It is also vitally important to ensure farmers’ safe return to work through placing currently unavailable testing and treatment facilities in their close vicinity.

## Supporting information

S1 File (XLSX)
